# Cul4A overexpression associated with Gli1 expression in malignant pleural mesothelioma

**DOI:** 10.1111/jcmm.12620

**Published:** 2015-07-27

**Authors:** Yi-Lin Yang, Jian Ni, Ping-Chih Hsu, Jian-Hua Mao, David Hsieh, Angela Xu, Geraldine Chan, Alfred Au, Zhidong Xu, David M Jablons, Liang You

**Affiliations:** a Department of Surgery, Helen Diller Family Comprehensive Cancer Center, University of California, San FranciscoSan Francisco, CA, USA; b Department of Oncology, Shanghai Pulmonary Hospital, Tongji University School of MedicineShanghai, China; c Department of Thoracic Medicine, Chang Gung Memorial HospitalTaoyuan, Taiwan; d Life Sciences Division, Lawrence Berkeley National Laboratory, University of California, BerkeleyBerkeley, CA, USA; e Division of Diagnostic Pathology, Helen Diller Family Comprehensive Cancer Center, University of California, San FranciscoSan Francisco, CA, USA

**Keywords:** malignant pleural mesothelioma, Cul4A, Gli1, hedgehog signalling, mTOR

## Abstract

Malignant pleural mesothelioma (mesothelioma) is a highly aggressive cancer without an effective treatment. Cul4A, a scaffold protein that recruits substrates for degradation, is amplified in several human cancers, including mesothelioma. We have recently shown that Cul4A plays an oncogenic role *in vitro* and in a mouse model. In this study, we analysed clinical mesothelioma tumours and found moderate to strong expression of Cul4A in 70.9% (51/72) of these tumours, as shown by immunohistochemistry. In 72.2% mesothelioma tumours with increased *Cul4A* copy number identified by fluorescence *in situ* hybridization analysis, Cul4A protein expression was moderate to strong. Similarly, Cul4A was overexpressed and *Cul4A* copy number was increased in human mesothelioma cell lines. Because Gli1 is highly expressed in human mesothelioma cells, we compared Cul4A and Gli1 expression in mesothelioma tumours and found their expression associated (*P* < 0.05, chi-square). In mesothelioma cell lines, inhibiting Cul4A by siRNA decreased Gli1 expression, suggesting that Gli1 expression is, at least in part, regulated by Cul4A in mesothelioma cells. Our results suggest a linkage between Cul4A and Gli1 expression in human mesothelioma.

## Introduction

Malignant pleural mesothelioma (mesothelioma) is a highly aggressive cancer that arises primarily from the pleural lining of the lung. The disease usually presents at an advanced stage and has a poor prognosis. To date, the mechanisms of mesothelioma pathogenesis have not been fully elucidated and there is no effective treatment. New therapeutic development is needed based on a greater understanding of mesothelioma’s underlying molecular mechanisms.

Cullin 4A (Cul4A), an evolutionally conserved cullin protein, provides a scaffold for ubiquitin ligases (E3) and functions in mediating proteolysis to regulate many cellular processes, including cell cycle, development, apoptosis and genome instability 1. Increased *Cul4A* copy number and Cul4A overexpression have been reported in various human cancers 2–5, and its oncogenic role has been reported *in vivo* and in mesothelioma cells 6,7. In human mesothelioma cells, down-regulation of *Cul4A* by shRNA induced cell cycle arrests in G0/G1 and inhibited the growth of mesothelioma cells 7. Although *Cul4A* overexpression has been suggested to promote growth of mesothelioma cells *in vitro*, the expression of Cul4A in mesothelioma tumour samples has not been studied.

In several cancers, Hedgehog (Hh) signalling has been implicated in the regulation of cell survival and proliferation, and Gli1 protein is one of the critical transcription factors that mediate the Hh signalling pathway. In recent studies of the importance of Gli1 expression in mesothelioma tumours, *Gli1* transcription and protein expression were increased significantly in mesothelioma tumours when compared to normal pleural tissues 8,9, and high *Gli1* expression was significantly associated with poor survival 9. Inhibition of Gli1 by siRNA or small molecular inhibitors was shown to suppress mesothelioma cell growth *in vitro* and in a xenograft model 8. Taken together, these studies suggested that Gli1 expression is important to the survival of mesothelioma cells.

In this study, we sought to determine whether Cul4A is overexpressed and/or amplified in mesothelioma tumours. To accomplish this, we analysed mesothelioma tumours and human mesothelioma cell lines using immunohistochemistry (IHC) and fluorescence *in situ* hybridization (FISH) analyses. We further studied the potential impact of increased Cul4A expression in mesothelioma cells. Because Gli1 expression was suggested to be critical to mesothelioma cell survival, we compared the protein expression of Cul4A and Gli1 in mesothelioma tumours and in mesothelioma cells. Furthermore, we analysed mammalian target of rapamycin (mTOR) and Gli1 expression after Cul4A inhibition, and a potential linkage between Cul4A, mTOR and Gli1 expression in mesothelioma cells was suggested in this study.

## Materials and methods

### Tissue samples, IHC and immunocytochemistry

Tissue microarray sections contained fresh mesothelioma and adjacent normal pleural tissues from patients with mesothelioma who were undergoing surgical resection of the primary tumour. Primary human mesothelioma samples from 73 patients were fixed in formalin and embedded in paraffin in 4-μm tissue microarray sections. In 10 of these patients, a small amount of normal pleural tissue had been obtained simultaneously to serve as controls. All human tissue samples were obtained and analysed in accordance with procedures approved by the institutional review board of the University of California, San Francisco (IRB H8714-22942-01).

The tissue microarray sections contained additional samples of the human mesothelioma cell lines MS-1, H290, H28, H2452, H226 and 211H. Histological sections of the tissue microarray were stained with haematoxylin and eosin for general morphology analysis. For IHC analysis, endogenous peroxidase was quenched for 15 min. at room temperature with 3% H_2_O_2_ in methanol in each lung section. Sections were blocked with 4% normal goat serum in PBS with 0.2% Triton for 2 hrs at room temperature before incubation overnight at 4°C with the properly diluted antibodies: anti-Cul4A (ab34897; Abcam, Cambridge, UK) at 1:400; anti-Gli1 (ab49314; Abcam) at 1:50. For immunocytochemistry (ICC) analysis, H2052 and LP-9 cells were fixed on glass slides using 5% acetic acid in ethanol for 2 min. Cell membrane was permeabilized using 0.25% Triton X-100 in PBS for 10 min. and endogenous peroxidase was quenched for 10 min. at room temperature with 3% H_2_O_2_ in PBS. Cells were blocked with 2% normal goat serum in PBS for 1 hr at room temperature before 1 hr incubation with the antibodies at room temperature.

Three independent researchers blindly scored positivity, and the data represent the samples that were scored positive by all three individuals. The following scoring system was used: −, no stain; +, weak staining (≥10% stained cellularity considered as positive); ++, moderate staining (≥30% stained cellularity considered as positive); +++, strong staining (≥50% stained cellularity considered as positive). All scoring was done under objective lens (×20) with a Zeiss Axioscop 2 microscope (Carl Zeiss, Jena, Germany) and photomicrographs were obtained with a Carl Zeiss AxioCam MrC5 camera under 20× or 40× objective lens.

### Cell culture

Human mesothelioma cell lines (MS-1, H28, H290, H2452, H226, 211H and H2052) and non-small cell lung cancer (NSCLC) cell line H1299 were purchased from American Type Culture Collection (Manassas, VA, USA). H28 pBABE Cul4A and H28 pBABE empty vector (EV) cell lines were prepared previously 7. All mesothelioma cell lines were cultured in RPMI 1640 medium supplied with 10% foetal bovine serum (FBS) and 1% penicillin-streptomycin. LP-9 cells were cultured using Ham’s F12 medium/Medium 199 (1:1 mixture) with 15% FBS, 2 mM L-glutamine, 1.7 nM epidermal growth factor and 1100 nM hydrocortisone. All the cells were cultured at 37°C and 5% CO_2_.

### Fluorescence *in situ* hybridization analysis

Fluorescence *in situ* hybridization analysis was performed on metaphase slides of mesothelioma cell lines, human mesothelial cell line LP-9, NSCLC cell line H1299 and human lymphocytes as the normal controls. The metaphase slides were probed with a bacterial artificial chromosome clone (RP11-391H12) targeting *Cul4A* at chromosome 13q34, as described previously 7. The bacterial artificial chromosome was labelled by nick translation with spectrum red deoxyuridine triphosphate and hybridized to metaphase slides overnight. A probe for centromere 13 labelled with spectrum green was purchased from Abbott, Inc. The chromosomes were counterstained with 4′,6-diamidino-2-phenylindole. Genomic copy numbers of *Cul4A* were determined by digital image microscopy after FISH.

### RNA interference

Cells were seeded in a 6-well plate as 50,000 cells/well with fresh media without antibiotics 24 hrs before transfection, with a target of 30–50% confluency at the time of transfection. *Cul4A* siRNA (ON-TARGET plus SMARTpool) and control siRNA were purchased from Thermo Scientific (Waltham, MA, USA). Cells were transfected with 100 nmol/l of siRNA using Lipofectamine RNAiMAX (Invitrogen, Carlsbad, CA, USA) according to the manufacturer’s protocol. After siRNA transfection, the plates were incubated for 48 hrs at 37°C before further analysis.

### Semi-quantitative RT–PCR

Total RNA from the various cell lines was isolated using the RNeasy extraction method (Qiagen, Valencia, CA, USA). First-strand cDNA was synthesized from total RNA by iScript cDNA synthesis (Bio-Rad, Hercules, CA, USA) according to the manufacturer’s instructions. Taqman RT–PCR analysis was performed on cDNA in a 384-well plate, using a Prism 7900HT Real-Time PCR System (Applied Biosystems, Foster City, CA, USA). Primers and Taqman probes for human *Cul4A*, human *Gusb* and human *Gli1* were purchased from Applied Biosystems. The expression of target gene in each sample was assayed in triplicate and normalized to human GUSB for mRNA expression analysis. Decreased transcriptional levels of Cul4A or Gli1 were calculated by dividing the transcriptional levels measured in Cul4A siRNA samples from those in the control samples.

### Western blotting

Mesothelioma MS-1 and 211H were seeded in a 6-well plate as 500,000 cells/well and cultured at 37°C supplied with 5% CO_2_ without antibiotics for 24 hrs. Cells were then transfected with Cul4A siRNA (siCul4A) or control siRNA (siCtrl). Cell lysates were then immunoblotted using anti-Gli1 (ab49314; Abcam), anti-mTOR (#2983; Cell Signaling, Beverly, MA) or anti-β-actin (#4970; Cell Signaling) antibodies. NIH ImageJ was used to quantify the intensity of western blots band, and the relative protein expression levels was calculated by normalizing with β-actin protein levels.

### Luciferase reporter assays

To measure Gli-mediated Hh transcriptional activity, the luciferase reporter constructs, 8× wild-type Gli binding site (8× Gli^wt^ Luc) or 8× mutant Gli binding site (8× Gli^mut^ Luc) plasmids 10, a human Gli1 expression vector (pcDNA3.1-Gli1), and Cul4A siRNA or control siRNA were co-transfected into NSCLC H1299 cells in a 24-well plate. The Renilla luciferase pRL-TK plasmid (Promega, Madison, WI, USA), whose expression is driven by the housekeeping thymidine kinase gene promoter, was co-transfected to normalize for transfection efficiency. All transfection experiments were performed using the Lipofectamine2000 (Invitrogen) in accordance with the manufacturer’s instructions. After 48 hrs cells were lysed and luciferase assays were performed as described previously 11. Results are expressed as fold induction, which is the ratio of luciferase activity induced in Gli-transfected cells relative to basal luciferase activity in control transfected cells. All experiments were performed in triplicate. Means and standard errors were calculated.

### Protein degradation assay

The stably transfected H28 pBABE (Cul4A overexpression) cells were treated with 100 μg/ml cycloheximide, the inhibitor of protein synthesis, and harvested at the time points of 0, 1 and 2 hrs. Total proteins were extracted and expression of mTOR was analysed by western blot.

### Statistical analysis

The data shown represent mean values ± SD. The chi-square independence test was used to compare IHC results between the staining intensity of Cul4A and Gli1 in the same mesothelioma tumours or cell lines. Student’s *t*-test was used to compare gene expression results and luciferase reporter activities between experimental and control groups. *P* < 0.05 were considered significant.

## Results

### Cul4A overexpression in mesothelioma tumours

**Figure 1:**
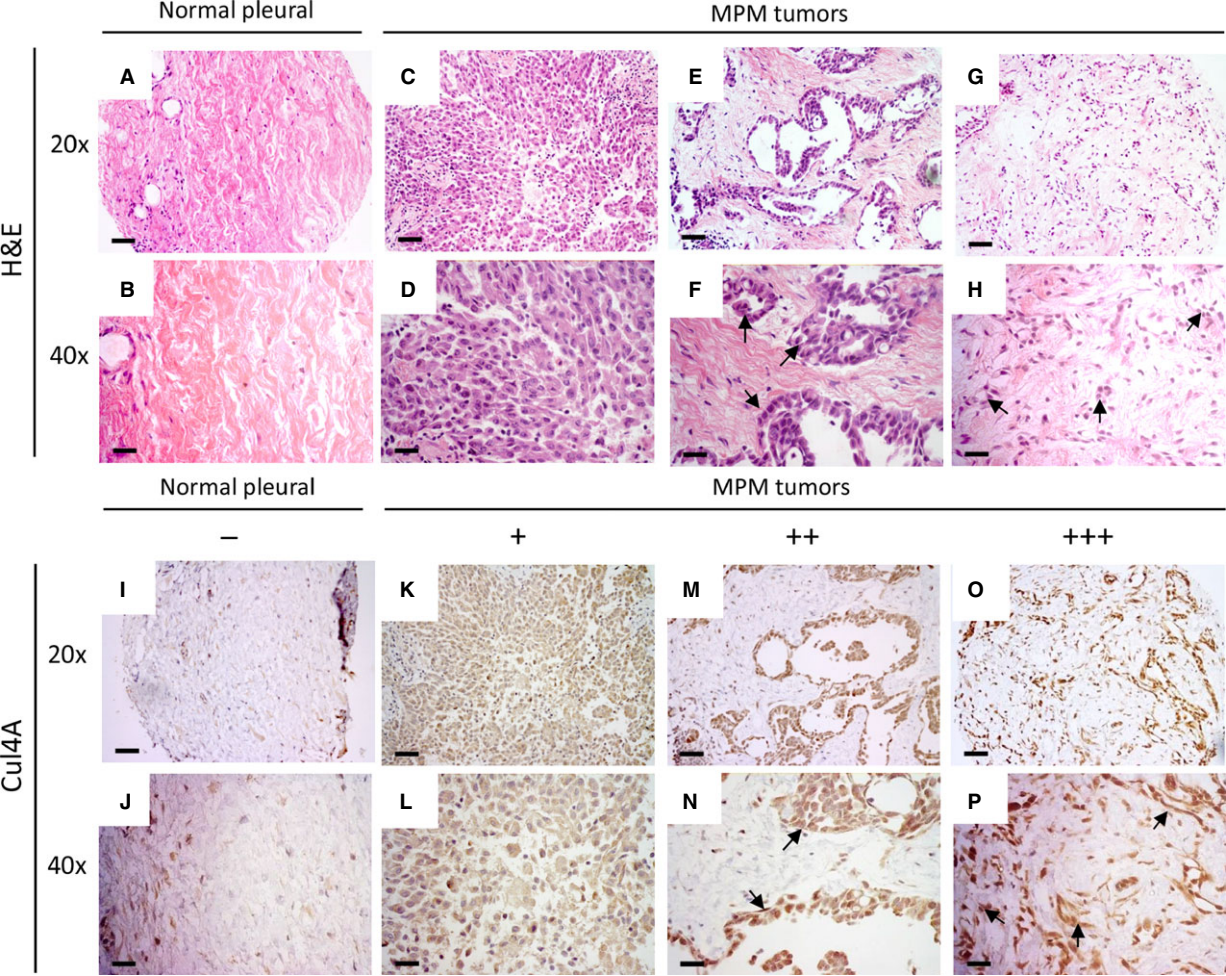
IHC analysis of Cul4A expression in mesothelioma tumours and control pleural tissues. Samples were stained with haematoxylin and eosin to show the morphology of the tumour cells in the tissue sections. Images from (A and B) normal pleural tissues or (C–H) mesothelioma tumours were captured under 20× or 40× objective lens. IHC analysis of Cul4A expression in normal pleural tissues was captured under (I) 20× or (J) 40× objective lens. Images of Cul4A expression in mesothelioma tumours with (K and L) weak Cul4A expression (M and N) moderate Cul4A expression, or (O and P) strong Cul4A expression were captured under 20× or 40× objective lens. Enlarged nuclei of tumour cells were shown by arrows (F, H, N, P; scale bar: 180 μm).

To investigate Cul4A expression levels in mesothelioma, primary human mesothelioma samples from 73 patients were analysed using IHC. Compared to the haematoxylin and eosin staining on normal pleural tissues (Fig. 1A and B), mesothelioma tumours showed enlarged cell nuclei under 20× (Fig. 1C, E and G) or 40× (Fig. 1D, F and H) objective lens. Among the 10 normal pleural tissues, Cul4A staining was negative in 50% (Fig. 1I and J), weak or moderate in 20%, and strong in 10% (Table 1). Among the 72 mesothelioma samples analysed (excluding one missing sample), Cul4A staining was negative in 9.7% (Table 1), weak in 19.4% (Fig. 1K and L) and moderate to strong in 70.9% (Fig. 1M and P). Cul4A staining was evident on the identical regions where the mesothelioma cells with enlarged nuclei were identified (Fig. 1N and P). From the clinical mesothelioma samples with available tumour phenotype information, 42 are epithelioid subtype, two are sarcomatous subtype and eight are biphasic (epithelioid/sarcomatous) subtype. There is no difference on the protein expression of Cul4A or Gli1, and Cul4A copy numbers detected between the subtypes of mesotheliomas analysed in this study.

**Table 1:** Cul4A expression in mesothelioma tumours and normal pleural tissues

Cul4A intensity from IHC	Tumour tissue (%) (*n*/total)	Normal tissue (%) (*n*/total)
−	9.7 (7/72)	50.0 (5/10)
+	19.4 (14/72)	20.0 (2/10)
++	40.3 (29/72)	20.0 (2/10)
+++	30.6 (22/72)	10.0 (1/10)

### Increased *Cul4A* copy number in mesothelioma tumour cells

**Figure 2:**
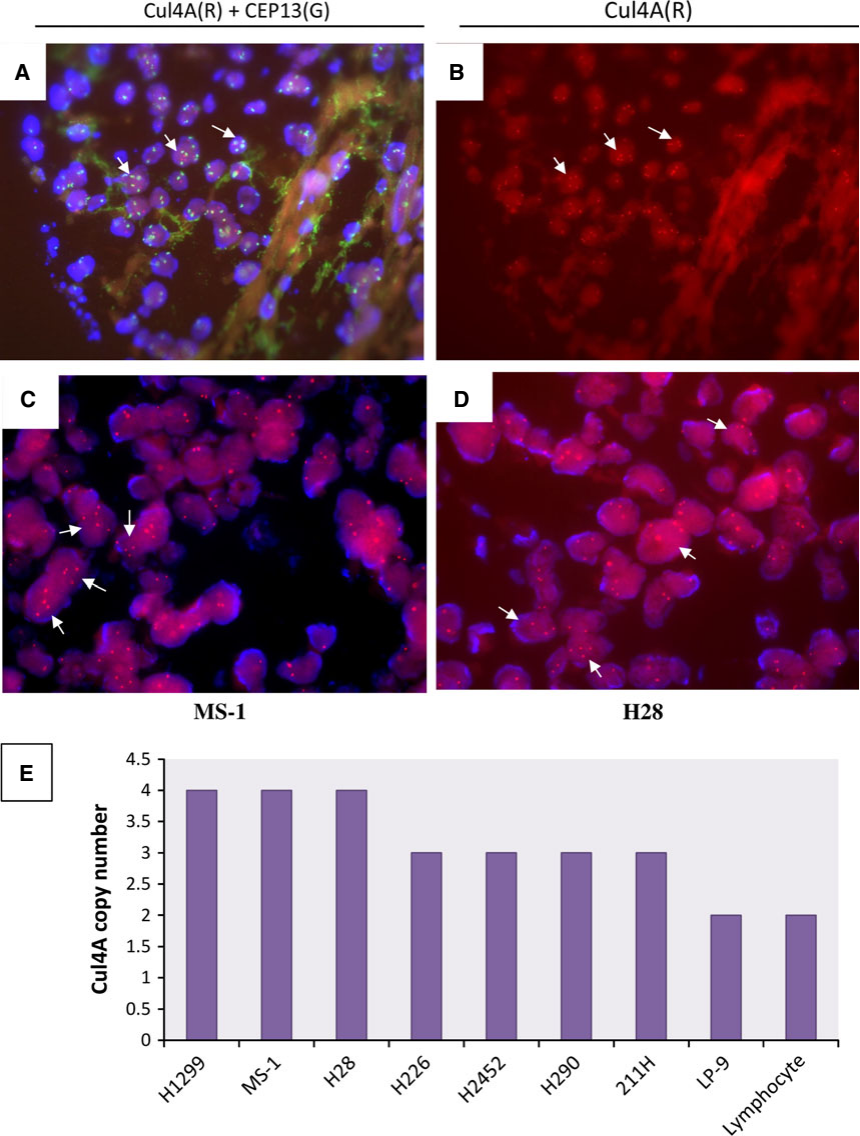
Increased Cul4A copy number in mesothelioma tumours and in human mesothelioma cell lines. Representative images of FISH assay for the copy number of the Cul4A gene on the same mesothelioma tumour showed (A) Overlaying signals from both Cul4A (red dots, arrows) and centromere 13 (green dots, arrows) probes, and (B) signals from only the Cul4A probe (red dots). Images of FISH assay for Cul4A copy number showed on mesothelioma (C) MS-1 or (D) H28 cells. The Cul4A probe is mapped on the chromosome 13q34 (red dots, arrows) and the centromere probe is mapped on the centromere region of chromosome 13. (E) A summary of Cul4A copy number detected by FISH assay on seven mesothelioma cell lines (MS-1, H28, H226, H2452, H290, 211H), mesothelial LP-9 cell line, NSCLC H1299 cell line and human lymphocytes.

To detect whether the *Cul4A* gene is increased in mesothelioma tumour samples, we measured the copy number of *Cul4A* using FISH. Among the 18 analysed mesothelioma tumour sections that showed 3–4 copies of *Cul4A* identified by FISH (Fig. 2A and B), Cul4A staining was moderate to strong in 13 samples when analysed by IHC. FISH also identified increased copy number of the centromere on chromosome 13 where the *Cul4A* gene is located (Fig. 2A). Our results showed a 72.2% concurrency of increased *Cul4A* copy number and Cul4A protein overexpression in the identical mesothelioma patient samples (Table S1), suggesting that the increased *Cul4A* copy number may contribute to the increased Cul4A expression in the mesothelioma tumours.

### Increased *Cul4A* copy number and Cul4A protein expression in mesothelioma cell lines

**Figure 3:**
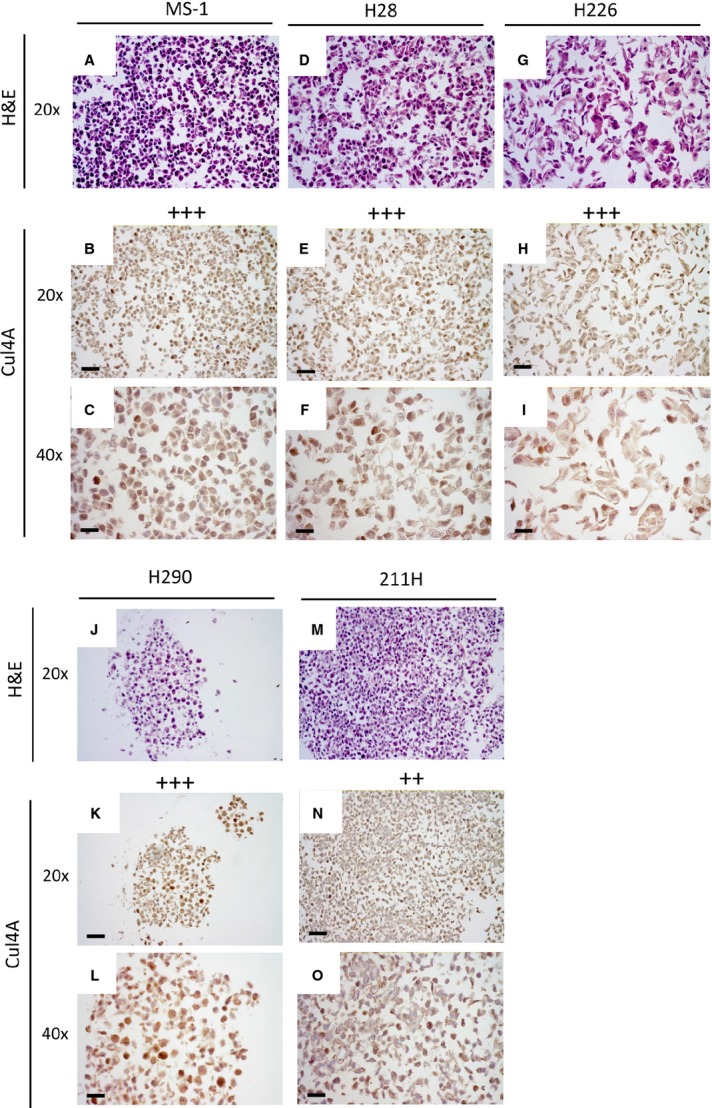
IHC analysis of Cul4A expression in human mesothelioma cell lines. Samples were stained with haematoxylin and eosin to show the morphology of mesothelioma cells (A) MS-1, (D) H28, (G) H226, (J) H290, (M) 211H. Images were captured under 20× objective lens. IHC analysis showed strong Cul4A expression in mesothelioma cell lines (B and C) MS-1, (E and F) H28, (H and I) H226 and (K and L) H290. Moderate Cul4A expression in (N and O) 211H cells were shown. Images were captured under 20× or 40× objective lens (scale bar: 180 μm).

We further analysed *Cul4A* copy number in seven human mesothelioma cell lines using FISH. This analysis showed 3–4 copies of *Cul4A* in mesothelioma cell lines MS-1 (Fig. 2C) and H28 (Fig. 2D), three copies in mesothelioma cell lines H226, H2452, H290 and 211H (Fig. 2E). Analysis of Cul4A copy number in NSCLC H1299 cells was included and H1299 cells showed four copies of Cul4A. Human mesothelial LP-9 cells and lymphocytes showed two copies of *Cul4A*. Since these results indicated that *Cul4A* copy number is increased in most of the analysed mesothelioma cell lines, we next analysed Cul4A protein expression in these cell lines using IHC. The enlarged cell nuclei in these cell lines were detected after haematoxylin and eosin staining, and Cul4A expression in these cell lines was shown under 20× or 40× objective lens (Fig. 3). Mesothelioma cell lines MS-1, H28, H226, H2452 and H290 showed strong staining of Cul4A, and 211H cells showed moderate staining. From the six mesothelioma cell lines analysed by IHC, four are epithelioid subtype and two are biphasic (sarcomatous/epithelioid) subtype. There is no difference on the protein expression of Cul4A or Gli1, and Cul4A copy numbers detected between subtypes of the mesotheliomas analysed in this study. In mesothelial cell line LP-9, Cul4A protein expression is minimal compared to that in mesothelioma cells 7. In addition, we analysed Cul4A and Gli1 protein expression in mesothelial LP-9 and mesothelioma H2052 cells using ICC (Fig. S1). Our results showed that the staining was negative (−) in the control samples without primary antibodies. Moderate staining (++) of Cul4A and Gli1 protein expression was detected in mesothelioma H2052 cells, and minimal staining (+) of Cul4A and Gli1 protein expression was detected in mesothelial LP-9 cells. Similar to our observations in mesothelioma tumours, six mesothelioma cell lines that had increased *Cul4A* copy number detected using FISH showed moderate to strong Cul4A staining by IHC, suggesting that increased *Cul4A* copy number may contribute to increased Cul4A expression in these cell lines.

### Positive association of Cul4A and Gli1 expression in mesothelioma tumours and mesothelioma cells

**Figure 4:**
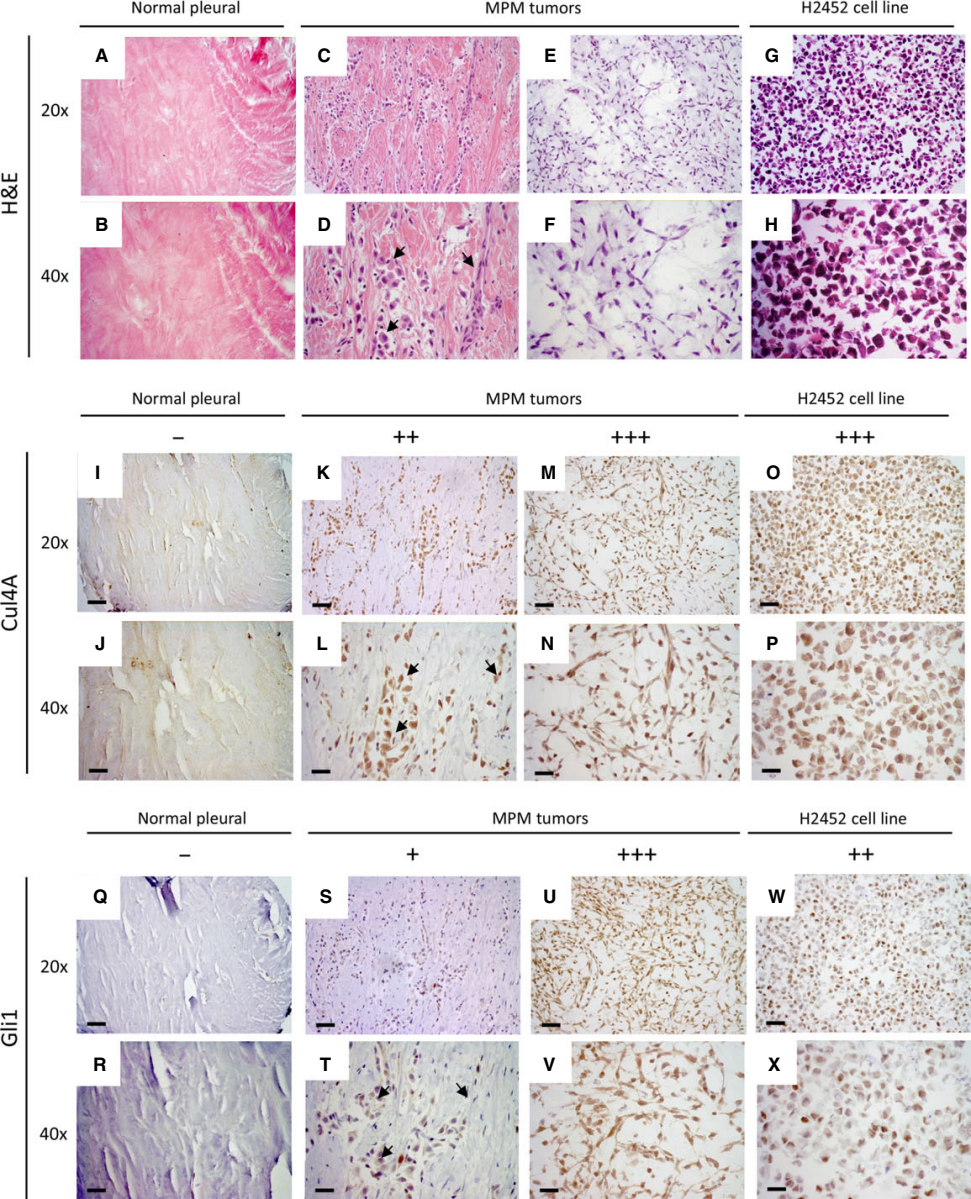
IHC analysis of Cul4A and Gli1 expression in mesothelioma tumours. Samples were stained with haematoxylin and eosin to show the morphology of the tumour cells in the tissue sections. Images from (A and B) normal pleural tissues, (C, D with arrows, E, F) mesothelioma tumours and (G and H) mesothelioma cell line H2452 were captured under 20× or 40× objective lens. IHC analysis of normal pleural tissues showed negative expression of Cul4A (I and J) or Gli1 (Q and R). Mesothelioma tumours with (K and L) moderate Cul4A expression to (M and N) strong Cul4A expression showed (S and T) weak Gli1 expression to (U and V) strong Gli1 expression. (L and T) Tumour cells are indicated with arrows. IHC analysis of mesothelioma H2452 cells showed (O and P) strong Cul4A expression and (W and X) moderate Gli1 expression. Images were captured under 20× or 40× objective lens (scale bar: 180 μm).

Since Gli1 expression is increased in mesothelioma tumours 9, we next analysed whether the Gli1 expression was related to Cul4A expression in the mesothelioma tumours. Compared to the haematoxylin and eosin staining on normal pleural tissues (Fig. 4A and B), mesothelioma tumours showed enlarged cell nuclei under 20× (Fig. 4C and E) or 40× objective lens (Fig. 4D and F). In normal pleural tissues, both Cul4A and Gli1 expression were negative (Fig. 4I, J, Q and R). In mesothelioma tumours, Cul4A and Gli1 expression were both increased (Fig. 4K, N, S and V) compared to that in the normal pleural tissues. The increased Gli1 expression was evident observed only in the cancer cells with enlarged cell nuclei (Fig. 4L and T, arrows), located in the identical regions where Cul4A was overexpressed. In addition, we compared Cul4A and Gli1 expression in seven human mesothelioma cell lines, including H2452 (Fig. 4G and H), using IHC. Among the six mesothelioma cell lines that had moderate to strong Cul4A expression (Fig. 4O and P, Table S1), four cell lines showed moderate to strong Gli1 expression (Fig. 4W and X). Our analysis of mesothelioma cell lines showed that Cul4A and Gli1 expression is associated, which is similar to our observation in mesothelioma tumours.

We next analysed the association of Cul4A and Gli1 protein expression in 71 mesothelioma tumours (excluding two missing samples) and seven mesothelioma cell lines (Table S1). Among the six mesothelioma samples with negative Cul4A expression, one showed negative Gli1 expression and two showed weak Gli1 expression (Table 2). Among 14 mesothelioma samples with weak Cul4A expression, 28.6% showed negative Gli1 expression and 50% showed weak Gli1 expression. Among 30 mesothelioma samples with moderate Cul4A expression, 33.3% showed weak Gli1 expression and 10.0% showed moderate or strong Gli1 expression. Among 28 mesothelioma samples with strong Cul4A expression, 42.9% showed moderate Gli1 expression and 14.3% showed strong Gli1 expression. Statistical analysis revealed a significant association of Cul4A and Gli1 expression in these mesothelioma samples (*P* < 0.05, chi-square test).

**Table 2:** Chi-square test for association analysis of Cul4A and Gli1 expression in mesothelioma tumours and cell lines (*P* < 0.05, chi-square)

	Cul4A (−) (%) (*n*/total)	Cul4A (+) (%) (*n*/total)	Cul4A (++) (%) (*n*/total)	Cul4A (+++) (%) (*n*/total)
Gli1 (−)	16.7 (1/6)	28.6 (4/14)	46.7 (14/30)	7.1 (2/28)
Gli1 (+)	33.3 (2/6)	50.0 (7/14)	33.3 (10/30)	35.7 (10/28)
Gli1 (++)	16.7 (1/6)	14.3 (2/14)	10 (3/30)	42.9 (12/28)
Gli1 (+++)	33.3 (2/6)	7.1 (1/14)	10 (3/30)	14.3 (4/28)

### *Cul4A* inhibition decreased Gli1 expression in mesothelioma cell lines

**Figure 5:**
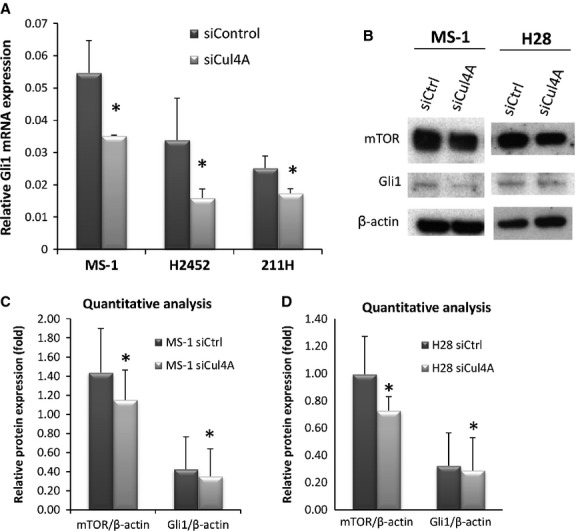
(A) Semi-quantitative RT-PCR analysis of *Gli1* mRNA expression in mesothelioma cell lines. *Gli1* mRNA expression was measured in mesothelioma cell lines MS-1, H2452, and 211H after *Cul4A* siRNA (dark grey bars) or control siRNA (light grey bars) treatment. The assay was done in triplicate and bars indicate SD. Asterisks * indicate *Gli1* transcription was significantly decreased when comparing to control samples. (B) MS-1 and H28 cells were treated with Cul4A siRNA (siCul4A) or control siRNA (siCtrl) for 48 hrs. Compared to the siCtrl control, MS-1 and 211H cells showed decreased expression of Gli1 mTOR after Cul4A knockdown. Quantitative analysis of western blotting bands showed decreased Gli1 and mTOR protein expression after siRNA treatment in (C) MS-1 and (D) H28 cells. Relative protein expression was calculated by normalizing the target protein expression with β-actin levels. The assay was done in triplicate and bars indicate SD. Asterisks * indicate relative Gli1 or mTOR protein expression was significantly decreased when comparing to control samples.

To further our understanding of the concurrent expression of Cul4A and Gli1 in mesothelioma samples, we investigated whether *Gli1* expression can be regulated by *Cul4A* levels in human mesothelioma cell lines. Three mesothelioma cell lines (MS-1, H2452 and 211H) with increased *Cul4A* copy number were treated with *Cul4A* siRNA for 48 hr and greater than 90% reduction in *Cul4A* mRNA was detected in all cell lines when compared to controls (*P < 0.05, *t*-test; Fig. S2A). When *Gli1* mRNA expression in the cell lysates was analysed using qRT-PCR, all the mesothelioma cell lines showed decreased *Gli1* expression levels after *Cul4A* knockdown (*P* < 0.05, *t*-test; Fig. 5A). *Gli1* mRNA was decreased by 53% in H2452 cells and by 31%-36% in 211H or MS-1 cells.

To confirm whether Cul4A positively regulates Hh/Gli1 signalling, we used luciferase reporter assay to detect the transcriptional activity of the pathway. NSCLC H1299 cell line with four copies of Cul4A (Fig. 2E) and mesothelioma cell line H290 was used in this analysis. Silencing of Cul4A by siRNA resulted in a significant decrease of Gli reporter activity in H1299 cells, compared with that treated with non-targeting siRNA (control; *P < 0.05, *t*-test). Inhibiting Cul4A by siRNA decreased Hh/Gli reporter activity in the mesothelioma H290 cells. The change was not significant (*P* = 0.05), which may due to the low basal level of Hh/Gli reporter activity in the mesothelioma cells (Fig. S2B).

Next, we used Cul4A siRNA to inhibit Cul4A expression and analysed Gli1 protein expression in mesothelioma MS-1, 211H and H28 cells using western blotting. Protein expression of Gli1 was decreased in all three cell lines after Cul4A knockdown (Fig. 5B, Fig. S2C). Because Cul4A has been shown to be involved in the ubiquitination and subsequent degradation of mTOR inhibitors 12,13, and mTOR was shown to regulate Gli1 expression through pathway crosstalk 14, we next measured mTOR expression in these cells. Our results showed that mTOR protein expression was decreased after Cul4A knockdown in MS-1 and H28 cell lines (Fig. 5B). Quantitative analysis of western blotting images showed that both Gli1 and mTOR protein levels were decreased after Cul4A knockdown in the mesothelioma cells (Fig. 5C and D, Fig. S2D). To further evaluate whether the expression of mTOR can be regulated by Cul4A in mesothelioma cells, we measured mTOR protein expression in the stably transfected H28 mesothelioma cells, which overexpress ectopic Cul4A gene 7. Increased Cul4A protein expression was detected in the Cul4A overexpressed H28 cells compared to the EV transfected control cells 7, and mTOR protein expression was significantly increased (3.5-fold) in the mesothelioma cells when Cul4A is overexpressed (*P < 0.05, *t*-test; Fig. S3A and B). We next tried to evaluate the role of Cul4A on the degradation of mTOR protein in Cul4A overexpressed H28 mesothelioma cells (pBABE Cul4A), following the addition of cycloheximide. In control siRNA treated cells, dramatic reduction of mTOR protein level was only showed at 8 hrs, which is consistent with the half-life of mTOR described by others 15. In Cul4A siRNA treated cells, dramatic reduction of mTOR protein level was observed at 2 hrs (Fig. S3C). Together, these results suggest that mTOR expression is, in part, regulated by Cul4A expression in the mesothelioma cells.

## Discussion

Our study shows that in mesothelioma tumours and human mesothelioma cell lines, Cul4A is overexpressed and *Cul4A* copy number is increased. Moreover, in 72.2% mesothelioma tumours with increased *Cul4A* copy number, Cul4A expression was moderate to strong. Therefore, increased *Cul4A* copy number appears to, at least in part, contribute to increased Cul4A expression in mesothelioma tumours and in mesothelioma cells. Cul4A expression also associated with Gli1 expression in mesothelioma tumours and in human mesothelioma cells, and inhibiting Cul4A expression by siRNA decreased Gli1 expression in mesothelioma cells. These findings suggest that Cul4A overexpression may contribute to Gli1 expression in the mesothelioma cells, and inhibiting Cul4A leads to Gli1 decrease in these cells. To further our understanding on the association of Cul4A and Gli1 expression in mesothelioma cells, we analysed mTOR expression after Cul4A knockdown by siRNA and mTOR expression was decreased. These findings suggest that mTOR pathway is involved in Cul4A-mediated Gli1 expression in mesothelioma cells.

Increased *Cul4A* copy number has been reported in several human cancers 1. We have previously shown that *Cul4A* copy number and protein expression are both increased in human mesothelioma cells 7. Inhibiting *Cul4A* expression by shRNA induces cell cycle arrest and decreases mesothelioma cell growth 7. In our *Cul4A* transgenic mouse model, lung tumorigenesis was observed after inducing *Cul4A* overexpression in the mouse lung, and inhibiting *Cul4A* expression by siRNA increased the sensitivity of lung cancer cells to chemotherapy drug cisplatin 6. Together, these studies suggest that increased *Cul4A* expression promotes tumour cell growth and is critical to tumour cell survival. Overexpressed Cul4A may also contribute to genomic instability by degrading replication licensing factor CDT1 in tumour cells 6. Increased Cul4A is positively correlated with distant metastasis in various human tumours 16. Cul4A was shown to promote migration and invasion of tumour cells *in vitro* and induce the growth and metastasis of tumour cells *in vivo* 16, suggesting that Cul4A can regulate epithelial-mesenchymal transition (EMT) and promote metastasis of tumour cells. Several signalling pathways are known to regulate EMT 17, includes Hh/Gli1 pathway. Whether the Hh/Gli1 pathway is involved in Cul4A-mediated EMT in tumour cells is unknown.

**Figure 6:**
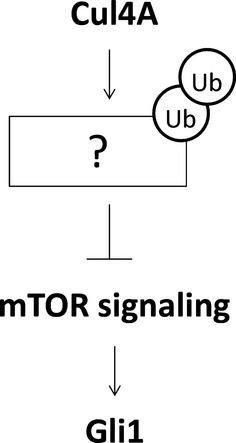
Schematic explanation of potential mechanism of Cul4A-mediated Gli1 expression in mesothelioma cells. Cul4A may target one of the mTOR inhibitors for degradation through ubiquitination (Ub) in mesothelioma cells, by which the mTOR pathway is activated. The activated mTOR pathway may have crosstalk with the Hh/Gli1 pathway, resulting in increased Gli1 expression in mesothelioma cells.

Although the regulatory mechanisms between Cul4A and Gli1 are not well-understood, Cul4A is known to mediate degradation of multiple components of the mTOR pathway 18. By associating with βTrCP, Cul4A has been shown to mediate proteolysis of REDD1, an inhibitor of mTOR signalling 13. By associating with FBXW5, Cul4A has been shown to mediate proteolysis of TSC2, another mTOR signalling inhibitor 12. In this study, we analysed mTOR protein level after Cul4A inhibition by siRNA, and our results showed that mTOR protein level was reduced faster in mesothelioma cells when Cul4A transcription was inhibited. These findings suggest that Cul4A overexpression could activate mTOR signalling by degrading its inhibitors. In addition, mTOR protein level in Cul4A siRNA treated cells was lower than that in the control siRNA treated cells at 0 hr, suggesting that mRNA level of mTOR could also be affected by Cul4A expression. Moreover, study of crosstalk between the mTOR and Hh pathways showed that activation of mTOR signalling increased *Gli1* transcription in human cancer tissues 14. A positive correlation between Gli1 and S6K1, an effector of mTOR signalling, was also reported 14. Studies have shown that mTOR signalling can activate Gli1 expression through canonical and non-canonical pathway in human cancer cells 14,19. When Gli1 was activated by mTOR signalling through the SMO-dependent canonical pathway, S6K1-mediated Gli1 phosphorylation prevents Gli1 from SuFu-mediated inhibition in the cancer cells 14. Moreover, the crosstalk between the pathways was further supported by evidence that combination treatments of SMO inhibitors and mTOR inhibitors showed synergistic effect on cancer cell inhibition 14,19. We have also observed decreased transcriptional activities of SMO and Gli1 in mesothelioma H28 cells after Cul4A knockdown by siRNA (data not shown), suggesting that SMO is in part involved in the Cul4A-mediated Gli1 expression. These findings suggest a potential mechanism that Gli1 expression increases as a result of Cul4A overexpression may be due to crosstalk of Hg/Gli1 pathway with the activated mTOR, induced by Cul4A-mediated proteolysis of mTOR inhibitors (Fig. 6). Further study is needed to evaluate this hypothesis and understand the mechanism that underlies the increase in Gli1 expression when Cul4A is overexpressed in human mesothelioma.

In this study, knocking-down Cul4A expression by using siRNA showed that Gli1 mRNA level and Gli1 protein level were both decreased in the treated mesothelioma cells, suggesting that inhibiting Cul4A decreased Gli1 expression. Our previous study has shown that Cul4A inhibition induces cell cycle arrests and reduces mesothelioma cell growth 7. In this study, our results showed that Cul4A inhibition reduces mTOR protein expression in mesothelioma cells. These findings suggest that Cul4A-mediated mesothelioma cell growth 7 may involve in elevation of Hh/Gli1 signalling through crosstalk between Hh and mTOR pathway. In cancer cells, there is crosstalk between Hh and other molecular signalling pathways, including rat sarcoma viral oncogene homolog (RAS)/ rapidly accelerated fibrosarcoma (RAF)/ mitogen-activated protein kinases (MEK)/ extracellular signal regulated kinases (ERK), PI3K/v-akt murine thymoma viral oncogene homolog (AKT)/mTOR, epidermal growth factor receptor (EGFR), and Notch 19. It is possible that other signalling pathways (*i.e*. RAS, EGFR, Notch) may also be involved in Cul4A-mediated tumour growth. Additional study is warrant to test this hypothesis.

The biological mechanism that promotes tumour cell growth by *Cul4A* overexpression is still under investigation, but one suggestion is that overexpressed Cul4A deregulates the cell cycle by degrading tumour suppressors p21 in mesothelioma cells 7. Cul4A has been shown to mediate ubiquitylation of p21, a cyclin-dependent kinase inhibitor 20,21. In our previous studies, we have shown that Cul4A down-regulation induces cell-cycle arrest by promoting p21 protein expression in mesothelioma cells 7 and overexpression Cul4A decreased p21 protein levels in a transgenic mouse model 6. Studies also shown that Gli1 inhibition induces cell-cycle arrest by promoting p21 transcriptional activity in human cancer cells 22,23. These findings suggest that Cul4A overexpression regulates cell cycle regulator p21 in cancer cells is possibly through Cul4A-mediated p21 protein degradation or through Cul4A-mediated Gli1 mRNA expression, in which p21 transcription was down-regulated.

The increased *Cul4A* copy number and its association with Gli1 regulation in mesothelioma cells highlight *Cul4A* as a possible new therapeutic target for mesothelioma tumours. Cul4A can be a potential target for developing anti-mesothelioma therapeutics because (*i*) Cul4A is amplified and overexpressed in mesothelioma tumours and cell lines as shown in this study and others 7, (*ii*) overexpressing Cul4A induces tumourigenesis in a mouse model 6 and (*iii*) knockdown of Cul4A expression reduces mesothelioma cell growth 7. In addition, targeting Cul4A presents low risk and minimal effects to patient health was also suggested 24. Moreover, a proteasome inhibitor Bortezomib (also known as Velcade or PS-341) that targets ubiquitin-proteasome system has been approved for the treatment of multiple myeloma and mantle cell lymphoma, and a small molecule MLN4924 that attenuates Cullin activity has entered phase I clinical trials for haematological and solid tumour malignancies 25,26. These findings suggest that Cul4A could be a target for developing anti-mesothelioma therapies. Targeting Cul4A in therapeutic development could decrease off-target effect and increase drug specificity to patients with Cul4A-amplified mesothelioma.

Our FISH analysis showed that some mesothelioma tumours with moderate to strong Cul4A expression showed two copies of *Cul4A* (Table S1). This may be an underestimate, given that (*i*) FISH analysis of clinical tissue sections was done in interphase, (*ii*) visualizing the locations of FISH probes in clinical tissue sections is difficult and (*iii*) 6/6 of mesothelioma cell lines showed increased *Cul4A* copy number (3–4 copies) by FISH (Fig. 2C and D). Another possibility is that the increased Cul4A expression in the mesothelioma tumours may be partially driven by other biological mechanism, which has not yet studied.

In conclusion, our results suggest Cul4A overexpression, driven by increased Cul4A copy number, in mesothelioma tumours and in mesothelioma cells. The increased Cul4A expression is associated with Gli1 expression in both mesothelioma tumours and in mesothelioma cells, and inhibiting Cul4A expression by siRNA decreased Gli1 expression in mesothelioma cells. Moreover, our results revealed that mTOR pathway is involved in Cul4A-mediated Gli1 expression in mesothelioma cells, possibility through mTOR activation by Cul4A-mediated proteolysis of mTOR inhibitors and through crosstalk between the activated mTOR pathway and Gli1 pathway. Furthermore, by revealing the Cul4A-mediated Gli1 expression in mesothelioma, our study provides a rationale for developing therapeutic agents targeting Cul4A in mesothelioma tumours.

## Supporting Information

**Figure S1** Immunocytochemistry analysis of Cul4A and Gli1 expression in mesothelioma H2052 and mesothelial LP-9 cells.

**Figure S2** (A) Semi-quantitative RT-PCR analysis of *Cul4A* mRNA expression in mesothelioma cell lines.

**Figure S3** (A) mTOR protein expression in Cul4A overexpressed (pBABE Cul4A) H28 cells and in control (pBABE EV) H28 cells was analysed using western blotting.

**Table S1** *Cul4A* DNA copy number was analysed using fluorescence *in situ* hybridization in 10 normal pleural tissues, 73 clinical mesothelioma tumours, seven human mesothelioma cell lines and one clinical normal lung tissue.

## References

[b1] Sharma P, Nag A (2014). CUL4A ubiquitin ligase: a promising drug target for cancer and other human diseases. Open biology.

[b2] Chen LC, Manjeshwar S, Lu Y (1998). The human homologue for the *Caenorhabditis elegans* cul-4 gene is amplified and overexpressed in primary breast cancers. Cancer Res.

[b3] Shinomiya T, Mori T, Ariyama Y (1999). Comparative genomic hybridization of squamous cell carcinoma of the esophagus: the possible involvement of the DPI gene in the 13q34 amplicon. Genes Chromosom Cancer.

[b4] Yasui K, Arii S, Zhao C (2002). TFDP1, CUL4A, and CDC16 identified as targets for amplification at 13q34 in hepatocellular carcinomas. Hepatology.

[b5] Abba MC, Fabris VT, Hu Y (2007). Identification of novel amplification gene targets in mouse and human breast cancer at a syntenic cluster mapping to mouse ch8A1 and human ch13q34. Cancer Res.

[b6] Yang YL, Hung MS, Wang Y (2014). Lung tumourigenesis in a conditional Cul4A transgenic mouse model. J Pathol.

[b7] Hung MS, Mao JH, Xu Z (2011). Cul4A is an oncogene in malignant pleural mesothelioma. J Cell Mol Med.

[b8] Li H, Lui N, Cheng T (2013). Gli as a novel therapeutic target in malignant pleural mesothelioma. PLoS ONE.

[b9] Shi Y, Moura U, Opitz I (2012). Role of hedgehog signaling in malignant pleural mesothelioma. Clin Cancer Res.

[b10] Sasaki H, Hui C, Nakafuku M (1997). A binding site for Gli proteins is essential for HNF-3beta floor plate enhancer activity in transgenics and can respond to Shh *in vitro*. Development.

[b11] Dhoot GK, Gustafsson MK, Ai X (2001). Regulation of Wnt signaling and embryo patterning by an extracellular sulfatase. Science.

[b12] Hu J, Zacharek S, He YJ (2008). WD40 protein FBW5 promotes ubiquitination of tumor suppressor TSC2 by DDB1-CUL4-ROC1 ligase. Genes Dev.

[b13] Katiyar S, Liu E, Knutzen CA (2009). REDD1, an inhibitor of mTOR signalling, is regulated by the CUL4A-DDB1 ubiquitin ligase. EMBO Rep.

[b14] Wang Y, Ding Q, Yen CJ (2012). The crosstalk of mTOR/S6K1 and Hedgehog pathways. Cancer Cell.

[b15] Fu L, Kim YA, Wang X (2009). Perifosine inhibits mammalian target of rapamycin signaling through facilitating degradation of major components in the mTOR axis and induces autophagy. Cancer Res.

[b16] Wang Y, Wen M, Kwon Y (2014). CUL4A induces epithelial-mesenchymal transition and promotes cancer metastasis by regulating ZEB1 expression. Cancer Res.

[b17] Lamouille S, Xu J, Derynck R (2014). Molecular mechanisms of epithelial-mesenchymal transition. Nat Rev Mol Cell Biol.

[b18] Zhao Y, Sun Y (2012). Targeting the mTOR-DEPTOR pathway by CRL E3 ubiquitin ligases: therapeutic application. Neoplasia.

[b19] Brechbiel J, Miller-Moslin K, Adjei AA (2014). Crosstalk between hedgehog and other signaling pathways as a basis for combination therapies in cancer. Cancer Treat Rev.

[b20] Abbas T, Sivaprasad U, Terai K (2008). PCNA-dependent regulation of p21 ubiquitylation and degradation *via* the CRL4Cdt2 ubiquitin ligase complex. Genes Dev.

[b21] Kim Y, Starostina NG, Kipreos ET (2008). The CRL4Cdt2 ubiquitin ligase targets the degradation of p21Cip1 to control replication licensing. Genes Dev.

[b22] Wang K, Pan L, Che X (2010). Gli1 inhibition induces cell-cycle arrest and enhanced apoptosis in brain glioma cell lines. J Neurooncol.

[b23] Srivastava RK, Kaylani SZ, Edrees N (2014). GLI inhibitor GANT-61 diminishes embryonal and alveolar rhabdomyosarcoma growth by inhibiting Shh/AKT-mTOR axis. Oncotarget.

[b24] Hannah J, Zhou PB (2013). The CUL4A ubiquitin ligase is a potential therapeutic target in skin cancer and other malignancies. Chinese journal of cancer.

[b25] Soucy TA, Smith PG, Milhollen MA (2009). An inhibitor of NEDD8-activating enzyme as a new approach to treat cancer. Nature.

[b26] Zhao Y, Sun Y (2013). Cullin-RING Ligases as attractive anti-cancer targets. Curr Pharm Des.

