# Supplementary figures and images for: Cul4A overexpression associated with Gli1 expression in malignant pleural mesothelioma
Source: J Cell Mol Med. 2015 Jul 27;19(10):2385–96. doi: 10.1111/jcmm.12620 (PMC4594680; doi:10.1111/jcmm.12620)

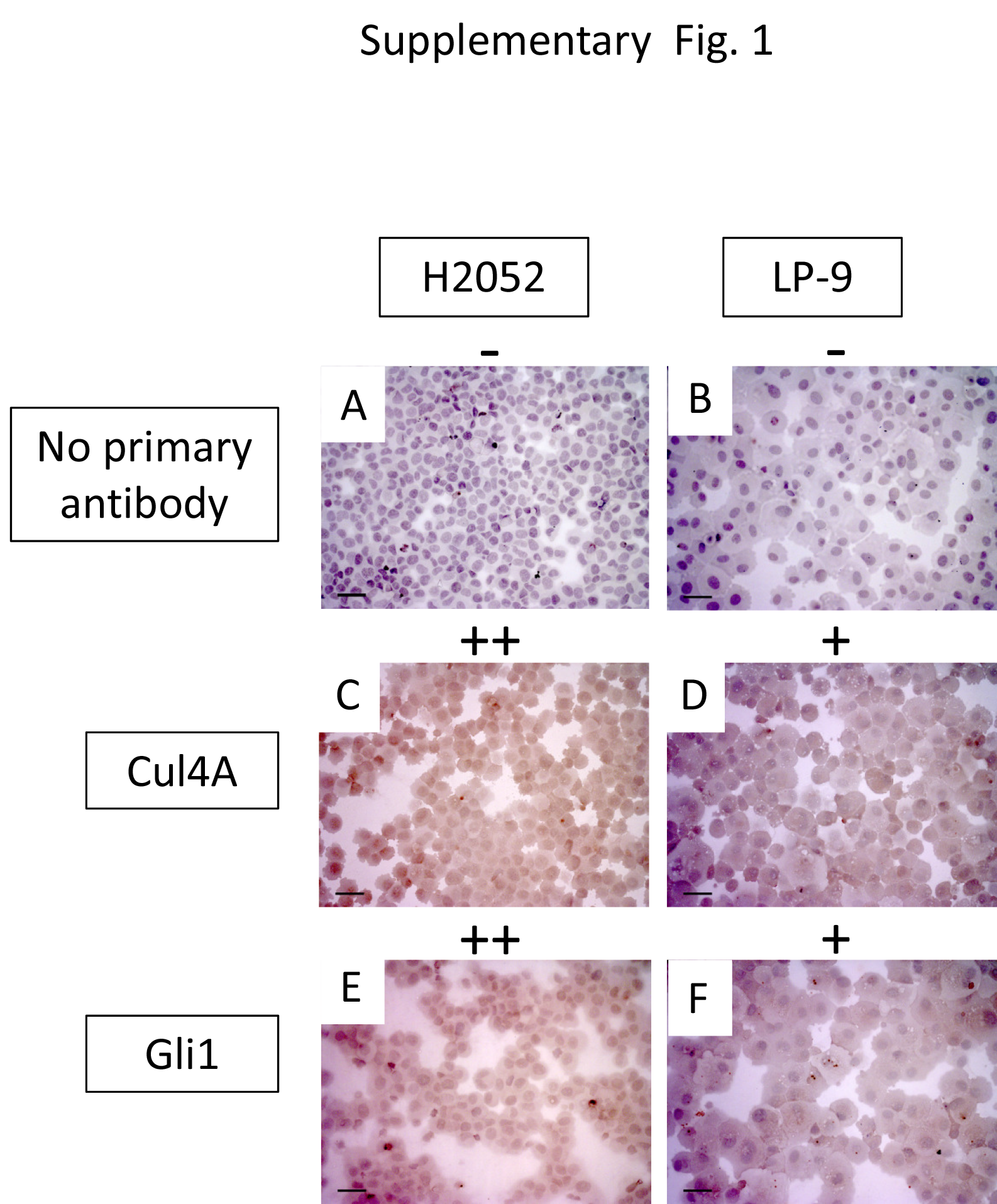

Supplement: Supplementary file 1 [file jcmm0019-2385-sd1.tif]

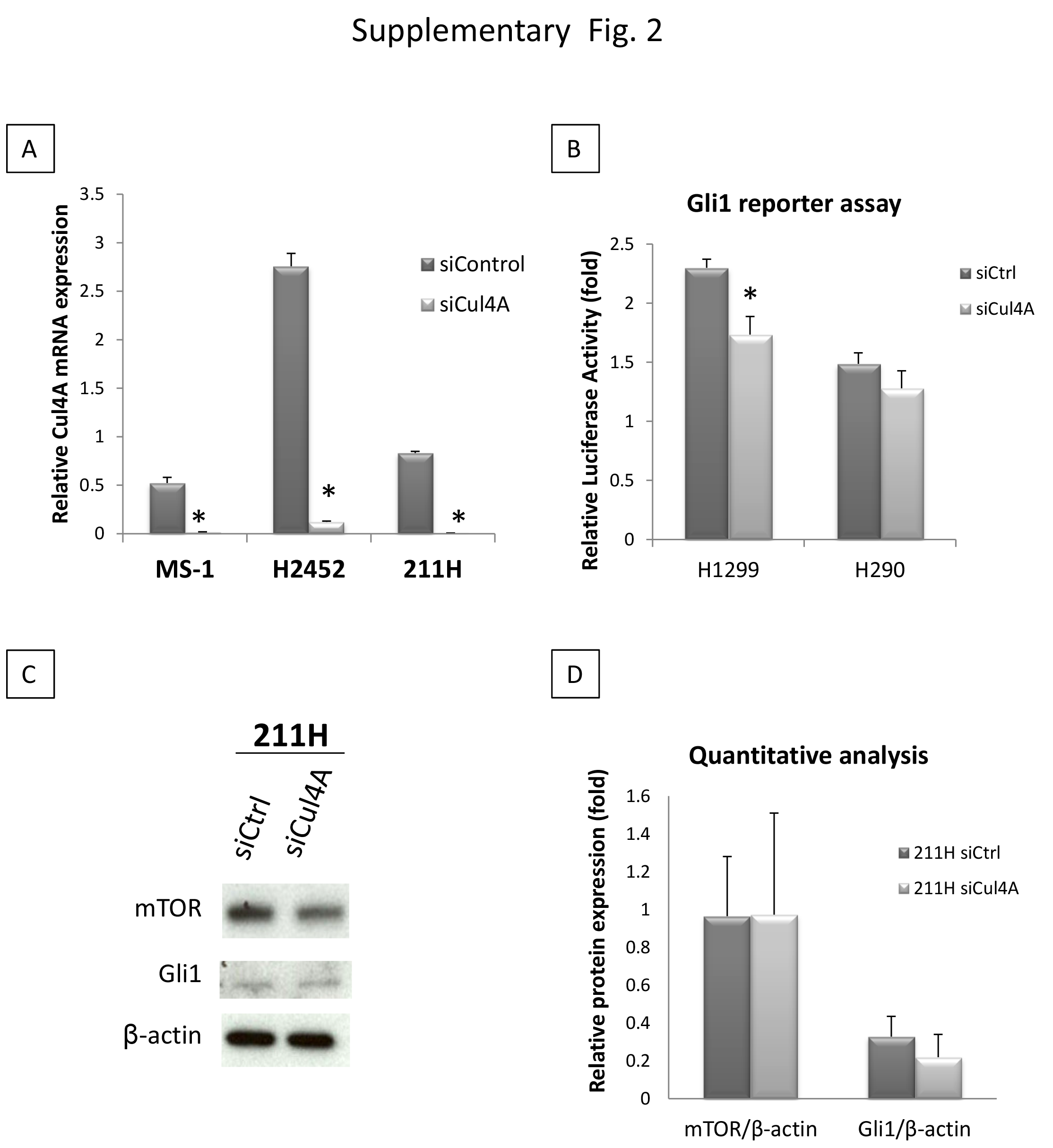

Supplement: Supplementary file 2 [file jcmm0019-2385-sd2.tif]

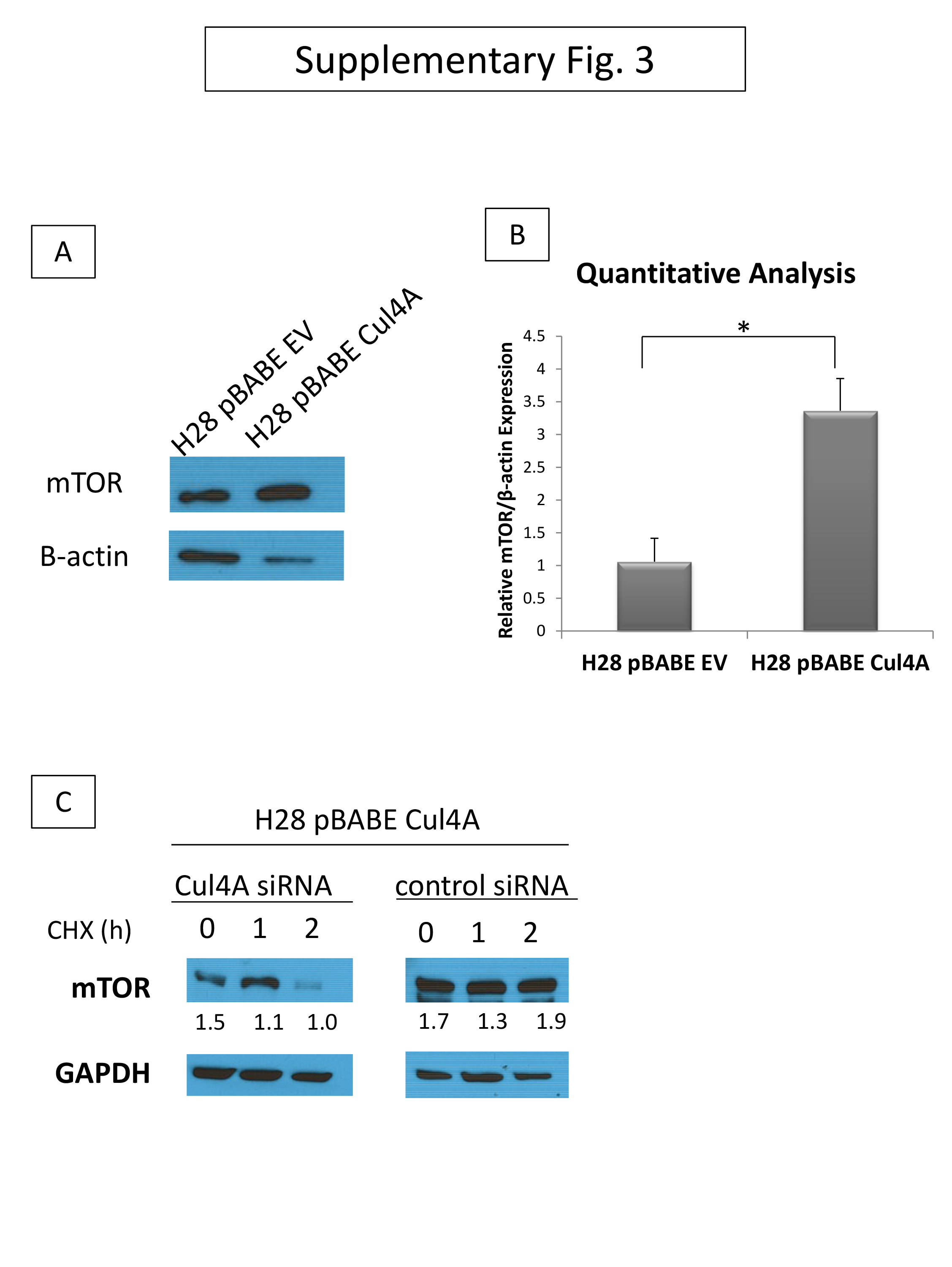

Supplement: Supplementary file 3 [file jcmm0019-2385-sd3.tif]
